# Submersible Spectrofluorometer for Real-Time Sensing of Water Quality

**DOI:** 10.3390/s150614415

**Published:** 2015-06-18

**Authors:** Adriana Puiu, Luca Fiorani, Ivano Menicucci, Marco Pistilli, Antonia Lai

**Affiliations:** Diagnostics and Metrology Laboratory, ENEA, Via Enrico Fermi 45, 00044 Frascati, Italy; E-Mails: luca.fiorani@enea.it (L.F.); ivano.menicucci@enea.it (I.M.); marco.pistilli@enea.it (M.P.); antonia.lai@enea.it (A.L.)

**Keywords:** light-induced fluorescence, spectrofluorometric sensor, water quality, chlorophyll detection

## Abstract

In this work, we present a newly developed submersible spectrofluorometer (patent pending) applied to real-time sensing of water quality, suitable for monitoring some important indicators of the ecological status of natural waters such as chlorophyll-a, oil and protein-like material. For the optomechanical realization of the apparatus, a novel conceptual design has been adopted in order to avoid filters and pumps while maintaining a high signal-to-noise ratio. The elimination of filters and pumps has the advantage of greater system simplicity and especially of avoiding the risk of sample degradation. The use of light-emitting diodes as an excitation source instead of Xe lamps or laser diodes helped save on size, weight, power consumption and costs. For sensor calibration we performed measurements on water samples with added chlorophyll prepared in the laboratory. The sensor functionality was tested during field campaigns conducted at Albano Lake in Latium Region of Italy as well as in the Herzliya Harbor, a few kilometers North East of Tel Aviv in Israel. The obtained results are reported in the paper. The sensitivity achieved for chlorophyll-a detection was found to be at least 0.2 µg/L.

## 1. Introduction

Gathering high-accuracy data with dense spatial and temporal distribution over large areas from lakes, seas and oceans is important for monitoring water pollution, improving water quality treatment, managing waste water and tracking climate change [1,2,3]. Moreover, it is also important for increasing sustainable and healthy agriculture via the implementation of advanced technologies for the monitoring of water contaminants [4].

Conventional analytical methods used for water quality analysis such as gas chromatography/mass spectrometry (CG/MS), high performance liquid chromatography (HPLC), enzyme-based systems, and flow cytometry are laboratory-based and require specialized personnel to carry out [5]. Moreover, they are expensive and require transportation of the sample to the laboratory, meaning there is a delayed response. Alternatively, high-speed detection instruments for real-time water monitoring based on mid-infrared [6], Raman [7] or fluorescence spectroscopy [8] have been developed in the last few decades. They have the advantages of being compact, non-destructive and less expensive with respect to the laboratory apparatus. Each of them is appropriate for rapid detection of specific water quality indicators with no or minimum sample preparation. A comprehensive description of the state-of-the-art of the technologies employed for water monitoring was reported by Hasan *et al.* in [9].

In this work, we report on developing a submersible sensor based on fluorescence spectroscopy  for real-time assessment of water quality at different depths. Fluorescence spectroscopy [10] is suitable to measure at least three of the most important indicators of the ecological status of natural waters: chlorophyll-a (Chl-a), oil, and protein-like materials. In fact, light-induced fluorescence (LIF) was widely used to detect both dissolved and particulate components of waters coming from aquifers, rivers and lakes [11,12,13,14,15,16,17,18,19,20,21,22,23]. Usually, for this kind of measurement, water undergoes a coarse filtration (30 μm), that eliminates unwanted materials, and the sample containing both dissolved and particulate components is pumped into a cuvette, where it is irradiated by one or more wavelengths. If only dissolved matter has to be analyzed, a fine filtration (0.22 μm) has to be carried out. Fluorescence spectra give information about the chemical composition of the sample (*i.e.*, carotenoids, phycoeritrin, phycocyanin, chlorophyll) [24]. Thus, each component has absorption and emission peaks allowing for its identification.

Spectrofluorometers are good candidates for a field campaign because, in general, they are battery-operated, fully controlled by a portable computer, and one measurement takes less than one minute. Once calibrated with known samples, they reach remarkable accuracies at absolute concentrations. Many submersible spectrofluorometers are available on the market [25,26,27,28,29], but they measure some selected wavelengths and not the full spectrum. This choice is probably dictated by economic reasons, but, from the scientific point of view, the availability of the full spectrum provides the instrument with unrivalled flexibility in different application scenarios and accuracy for many water components. In a recent publication, MacIntyre *et al.* [17] indicated that the sensitivity for Chl-a of LED based commercial instruments is <1 µg/L. Our team’s experience with laser-based systems for fluorescence detection has allowed us to reach a sensitivity of about 0.1 µg/L for Chl-a [30]. This better sensitivity is not surprising because optical power is higher in a laser than in an LED.

Based on these considerations, we focused on developing a low-cost submersible sensor for non-invasive, real-time sensing of water quality (patent pending) able to measure fluorescence in a wide spectral interval (200 nm–1100 nm) and to detect at least 0.2 µg/L of Chl-a by using LEDs instead of lasers. The achieved sensitivity lies in between LED-based commercial instruments and laser systems, as can be explained by the detection of the full spectrum (compared to commercial instruments) and the lower optical power (compared to laser systems). In the following sections, a thorough presentation of the instrument components, calibrations, laboratory tests and results of field campaigns will be reported.

In the future, it will be possible to build a network of submersible spectrometers able to communicate and transfer measurements by optical wireless links to an analysis center able to integrate spectrometer network data with satellite data and fuse them in order to get hyper-precise results.

## 2. Instrument Design

The submersible spectrofluorometer was designed by building on our team’s experience in remote and local LIF sensors. The previous patented system used in a number of campaigns in the Mediterranean Sea [31], inland waters [32], the St Lawrence Estuary [33] and the Arctic Ocean [34] was based on double laser excitation in the UV (266 nm) and visible (405 nm) spectral region and double water filtration. The advances in the new instrument compared to the old patented one are highlighted by the following features: it is submersible; it is based on light-emitting diodes (LEDs) instead of lasers; it avoids the use of filters and pumps; being submersible, it can immediately characterize water at different depths till 20 m, but there are no limitations to realize a chamber that allows for deeper immersion.

The elimination of filters and pumps was decided on in order to increase system simplicity and to avoid the risk of sample degradation. Of course, this solution could allow some solar radiation to reach the sensor, but this issue has been circumvented by a specially designed screen.

### 2.1. Light Sources and Spectrometer

In a fluorescence spectroscopy experiment, various light sources may be used as an excitation source: lamps (*i.e.*, xenon arcs and mercury-vapor lamps), lasers or light emitting diodes (LEDs). Lamps can have serious disadvantages compared to lasers and LEDs such as lack of monochromaticity, heat generation, cost and short lifetime. The only advantage of lamps is the availability of a broadband spectrum, which is not mandatory in our case because just a few wavelengths are to be used. Instead, lasers and LEDs are monochromatic or near monochromatic, less expensive, produce little heat, are small, and have an extended lifetime. Among all the light sources, LEDs are more compact and less expensive. Moreover, they have the advantage of long life and low power consumption while lasers and lamps require a high voltage power supply. Keeping in mind that a submersible device has to be battery operated, LEDs turned out to be the ideal candidates. The last five years of development in LED technology allow the production of high radiant power LEDs in the visible range, from 460 nm to 645 nm.

The choice of the LEDs wavelengths was guided by the absorption characteristics of the three important indicators of the ecological status of natural waters we want to monitor: Chl-a, oil, and protein-like materials (tyrosine and tryptophan). Typical absorption and emission bands of these substances are listed in Table 1. The Chl-a level in water is a direct way of tracking algal growth and an indirect indicator of nutrient levels, while protein-like material and oil are key markers of biological and chemical pollution of water.

**Table 1:** Absorption and emission bands of some important indicators of the ecological status of coastal waters.

Indicator	Absorption (nm)	Emission (nm)
Protein-like material (tyrosine and tryptophan)	270–290	350–400
Oil	280–300	350–400
Chl-a	400–500	670–690

In order to detect these three substances, a miniature spectrometer and two kinds of LEDs emitting 280 nm and 450 nm, respectively, were used for building the prototype. LEDs emitting 280 nm greatly improved the fluorescence yield of protein-like material and oil, while LEDs emitting 450 nm excited Chl-a fluorescence. Although we focused on the three components of Table 1, the availability of the full emitted spectrum allows the sensor to detect many other pollutants and pigments and, as a consequence, to be deployed in various water bodies with different ecological statuses.

**Figure 1:**
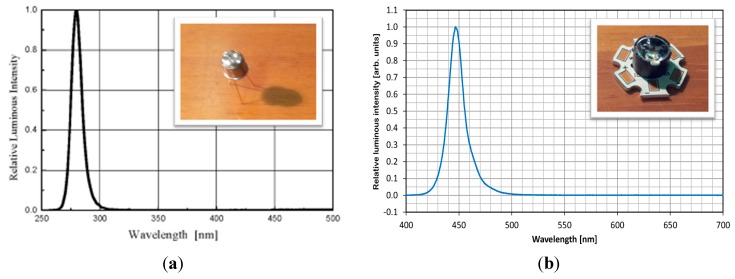
LEDs used for fluorescence excitation: (**a**) UV-LED emitting 280 nm; (**b**) blue LED emitting 450 nm.

After careful market research, the following items were chosen as they fulfilled specific requirements (*i.e.*, cheap, compact, low energy consumption): (a) “USB2000+” Spectrometer (detector range: 200–1100 nm, optical resolution: 0.3 nm by Ocean Optics, Winter Park/USA); (b) Seoul Semiconductor “T9B28C” LED with ball lens included (Ansan/Korea), emitting 600 μW at 280 nm with ±5° full with at half maximum (FWHM) emission angle (Figure 1a); (c) Osram Opto Semiconductors Oslon SSL “Deep Blue” LED (Regensburg/Germany) equipped with a “Lisa2-Pin Real Spot” lens, emitting 560 mW at 455 nm with ±8° FWHM emission angle (Figure 1b).

**Figure 2:**
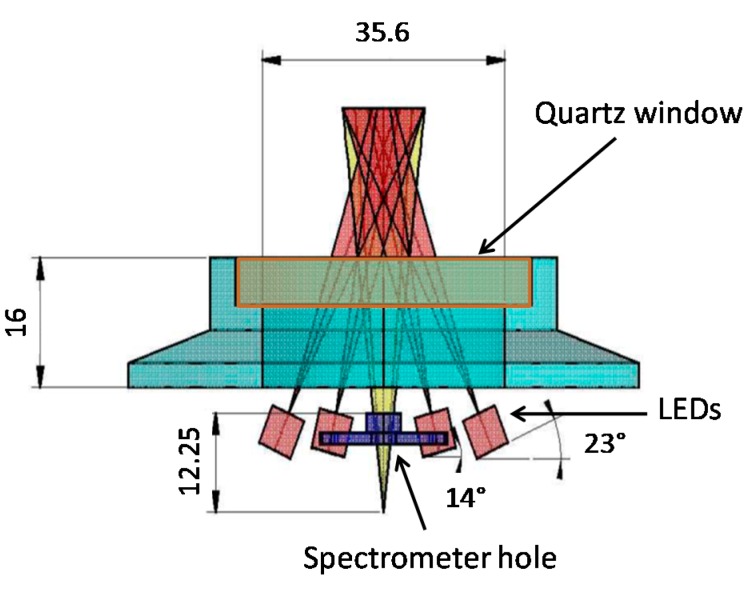
The water region observed by the spectrometer is illuminated by the UV and blue LEDs. Water and optoelectronic devices are separated by a quartz window (NHI-1191 by Helios Italquartz, Milan/Italy) with transmittance greater than 90%.

With the configuration reported in Figure 2, 2.4 mW of UV light (coming from 4 LEDs) and 1.12 W of blue light (coming from 2 LEDs) are delivered in the water region observed by the spectrometer. Laboratory tests showed that the sensitivity of the latter is so high that its charged-coupled device (CCD) is saturated by the tiny out-of-band LED emission. In order to avoid this effect, we filtered the LED emission. This approach reduces the emitted power but allows one to observe all the wavelengths below the cutoff wavelength. Keeping in mind that blue LEDs have relatively high power, a Semrock 447/60 BrightLine^®^ Bandpass Filter, New York/USA (realized according to our specifications) was placed in front of each blue LED.

### 2.2. Mechanics and Electronics

In the design of the mechanical part, emphasis was put on robustness and resistance to high external pressures. For this, prior to assembling the spectrometer, the electronics and the optical components inside the submersible chamber, the following compliance tests were performed: (a) a leak test with He; (b) mechanical resistance and air-tightness test; (c) check of the safety valve functionality; (d) pressure test with N_2_.

**Figure 3:**
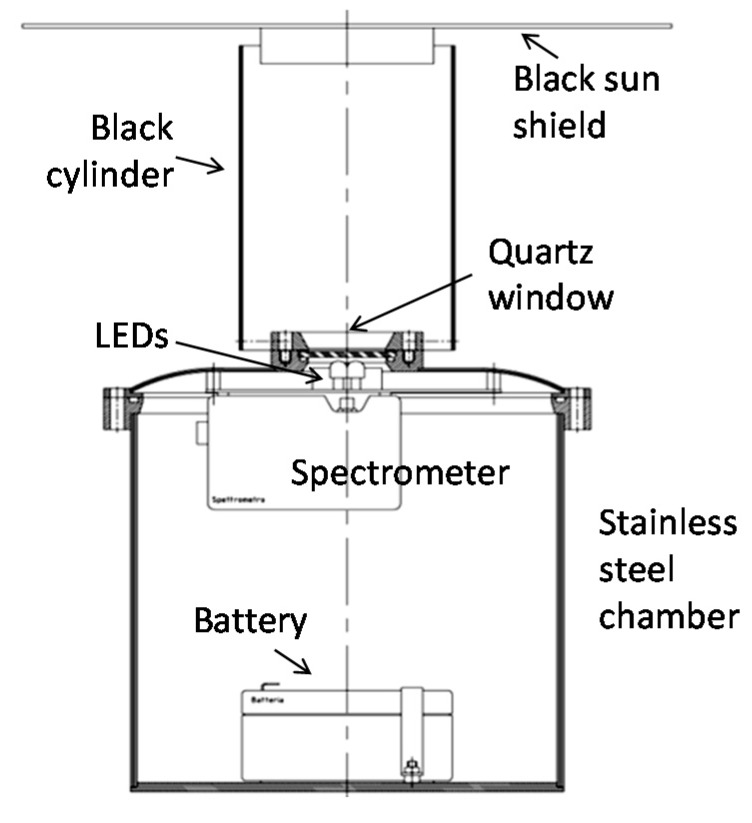
Technical drawing of the external chamber.

A technical drawing of the chamber configuration is presented in Figure 3. As can be seen, a cylinder ending with a sun shield is placed on the chamber cover. This cylinder will be filled with water when the instrument is submerged, thus allowing an analysis of the sample without any interference from the external light or the need for filters and pumps.

**Figure 4:**
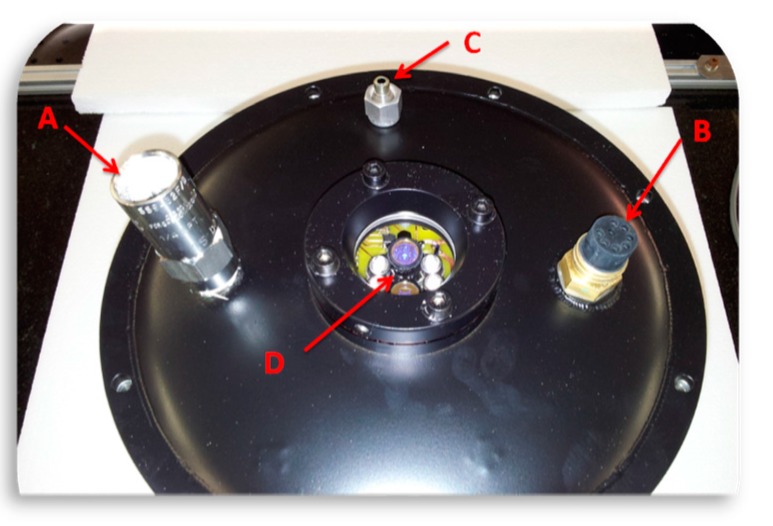
View of the chamber cover: A—safety pressure valve; B—electrical connector; C—gas inlet; D—quartz glass window covering the LEDs and the spectrometer.

The chamber cover is provided with a safety valve and a connector (Micro WER-CON 8 poles waterproof connector by Seacon, El Cajon/USA) for both data transmission and battery charging (Figure 4).

**Figure 5:**
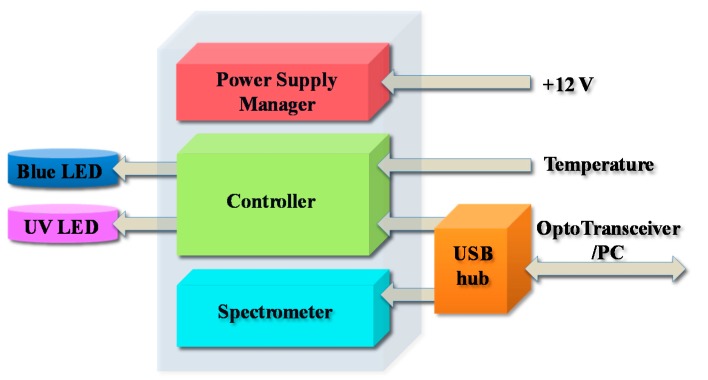
Electronic layout of the sensor.

The electronic layout of the sensor is given in Figure 5. At this time, the sensor is linked to the personal computer (PC) by a USB cable, while in the future this connection will be ensured by an optical transceiver. The electronic card performs the following operations: controls (on/off) the four UV LEDs, controls (on/off) the two blue LEDs, reads the board temperature, reads the box temperature (proxy of the water temperature), reads the battery voltage, and sends/receives data to/from the PC through USB.

**Figure 6:**
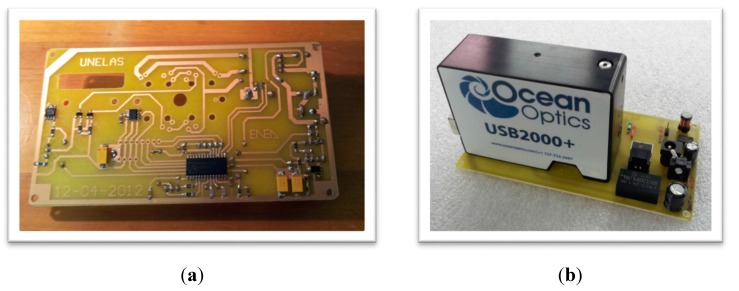
Detail of the electronic assembly: (**a**) printed circuit board; (**b**) assembled sensor.

An image of the printed circuit board (PCB) is presented in Figure 6a, while the complete electronic assembly can be seen in Figure 6b. The main components of the electronic card are: (a) microcontroller unit (MCU)—microchip PIC18F2550, firmware developed in PicBasicPro and Assembler; (b) temperature sensor—TCN75-5.0, digital output, I2C bus; (c) voltage of the rechargeable battery read by the MCU internal analog-to-digital converter (ADC); (d) switched-mode power supply (SMPS)—MC34063A-step-down as power supply (+5 V) with 11 V ≤ V_battery_ ≤ 14 V); (e) a sealed lead-acid, 12 V, 2.3 Ah battery.

A Visual Basic user-friendly graphical user interface (GUI) has been developed in order to control and monitor the sensor from the PC.

### 2.3. Assembled Prototype

**Figure 7:**
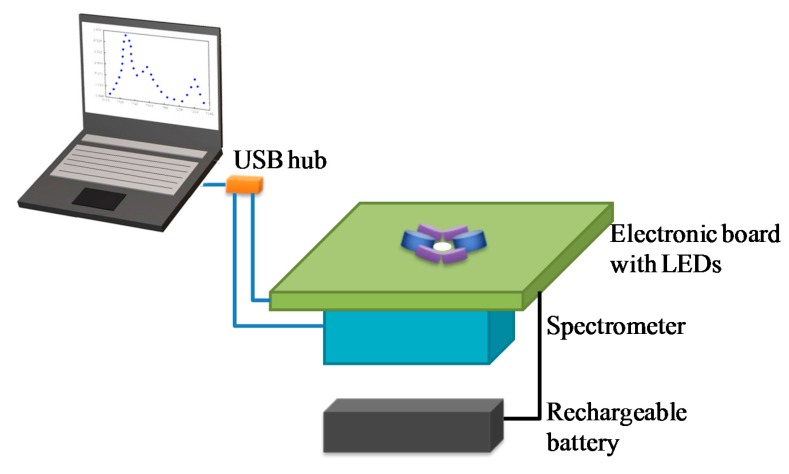
Instrument block diagram.

The chosen components previously described were assembled following the scheme depicted in Figure 7. The spectrometer and the electronic board with the LEDs and optics were placed in the stainless steel waterproof container (submersible chamber) at atmospheric pressure.

**Figure 8:**
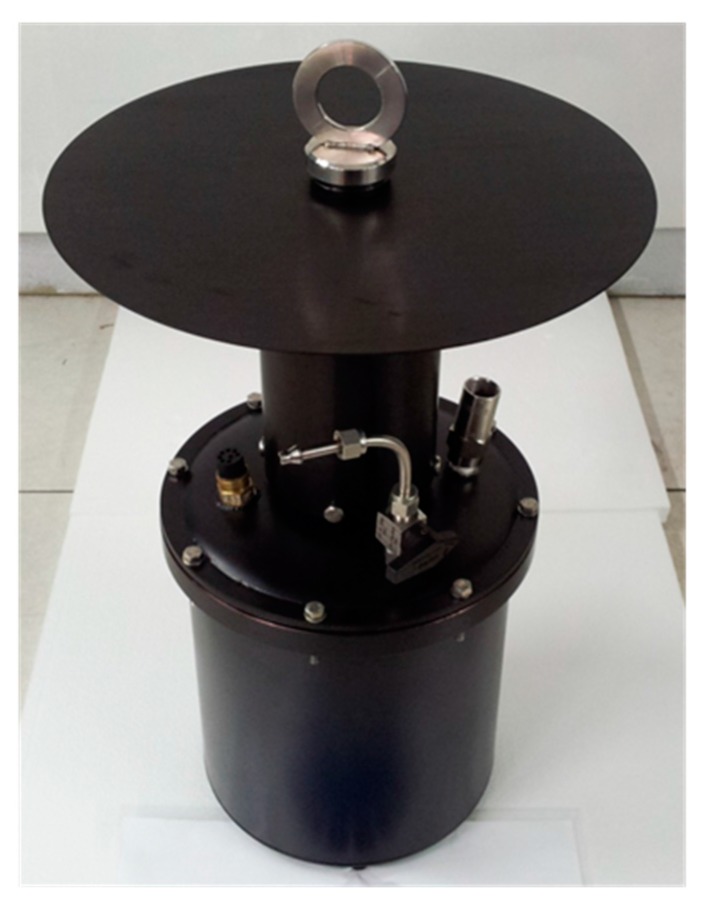
Image of the assembled prototype.

A black flat cover shielding the sensor from solar radiation was mounted. The black cylindrical screen minimized the scattered radiation at the window. An overview of the assembled submersible instrument is given in Figure 8.

(280 nm) followed by focusing lenses (model Lisa2-Pin-OSL miniature lenses by LEDil) in order to reduce their emission angle; (b) blue LEDs (450 nm) covered by bandpass filters (model BrightLine^®^ by Semrock) in order to suppress the LED radiation far from the emission peak, resulting in a reduction of the emission bandwidth; (c) spectrometer detecting the fluorescence spectrum from the middle UV (200 nm) to the near infrared (IR) (1100 nm) with high resolution (0.3 nm); (d) waterproof box containing LEDs, spectrometer and related electronics, with a waterproof window transmitting from the middle UV to the near IR.

In summary, the submersible spectrofluorometer components are as follows: (a) middle UV LEDs The spectrometer measures simultaneously and in real-time the fluorescence spectra excited by UV and blue LEDs in the natural waters where it is immersed. The fluorescence spectra provide simultaneous and real-time levels of Chl-a and pollution (protein-like material and oil) in the natural waters, thanks to a specially developed acquisition software. The instrument design allow water to be probed without any filter or pump and the specially designed shields (flat cover and cylindrical screens) offer protection from solar radiation by preventing its interference with the measurement.

### 2.4. Hardware and Software for Data Acquisition and Processing

Data acquisition and processing was entrusted to a convertible notebook which sets the benchmark for ruggedness: vibration- and shock-resistant, water- and dust-resistant (model “Toughbook CF-19” by Panasonic, Newark, NJ, USA) and with an integrated GPS.

**Figure 9:**
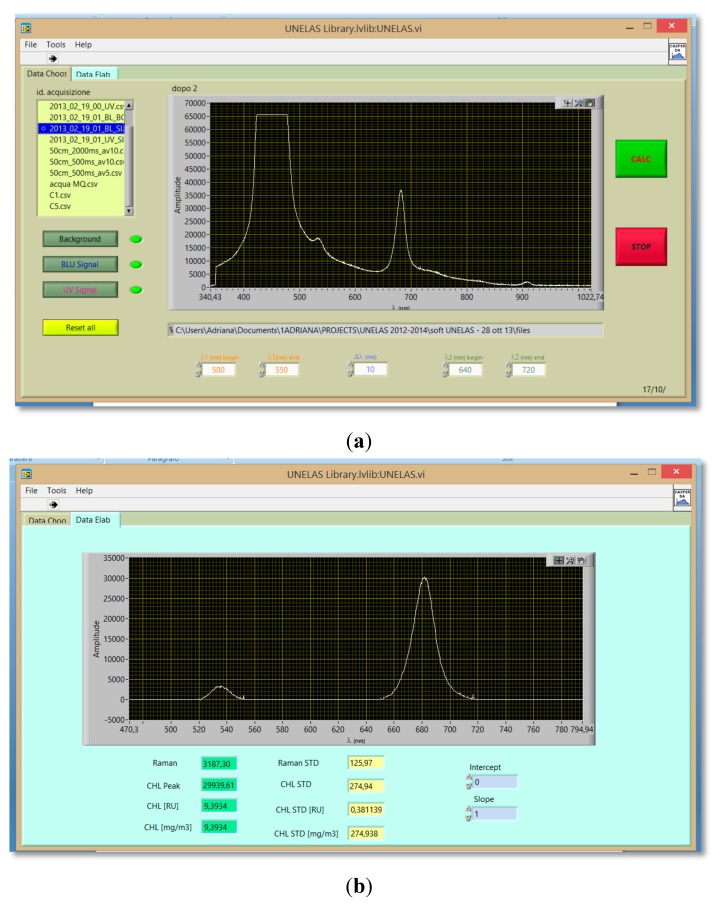
Panel of the data processing software showing: (**a**) unprocessed spectra; (**b**) results of the automatic data processing.

A friendly graphical user interface was developed in the LabView environment for rapid data processing. In the first panel of the developed application the user can load the data to be analyzed and set the calculus parameters. A second panel shows the background-free spectrum and all calculated results (Figure 9).

The developed data processing software calculates Chl-a concentration, standard deviation and measurement error over a certain number of acquisition points around the peak maximum, which can be fixed by the user.

## 3. Materials and Procedures

### 3.1. Materials and Procedures for Laboratory Tests

For the calibration in laboratory, we prepared one reference sample with pure Milli-Q water and five samples (C1, C2, …, C5) of Milli-Q water containing different concentrations of Chl-a (supplied by Sigma-Aldrich) ranging from 10 µg/L to 200 µg/L. The Chl-a standard was previously dissolved in pure acetone (100%). The most concentrated sample C1 was subsequently diluted by adding different quantities of Milli-Q water, in order to obtain the less concentrated samples C2, C3, …, C5. All the spectra were acquired by using 200 ms integration time and an average of 10 acquisitions. The calibration was performed by correlating the data acquired by the sensor with the chlorophyll measurement by a commercial Spectrometer (model *Lambda 25UV/VIS* by PerkinElmer, Waltham, MA, USA).

The statistical error E is calculated over n = 10 acquisition points around the peak maximum with the following Equation (1):

(1) $$E=\frac{\text{STD(Chl)}}{\sqrt{n}}$$

where the standard deviation of the Chl-a measurement—STD(Chl) is automatically calculated by the specially developed software, following the rule for error propagation Equation (2):

(2) $$\text{STD(Chl)}=\text{ R}\sqrt{{\left(\frac{STD(I_{Chl})}{I_{chl}}\right)}^{2}+{\left(\frac{STD(I_{Raman})}{I_{Raman}}\right)}^{2}}$$

where R = I_chl_/ I_Raman_ is the Chl-a concentration expressed in Raman Units [RU] _,_ I_chl_ is the Chl-a fluorescence peak intensity, I_Raman_ is the water Raman peak intensity, STD(I*_chl_*) and STD(I*_Raman_*) are the standard deviations calculated over 10 acquisitions around the maximum peak value.

The value of the statistic relative error E_R_ is calculated with the Equation (3):

(3) $$E_{R}=\frac{\text{E}}{Chl(\mu g/l)}F$$

where F is the sensor calibration factor, and Chl (µg/L) is the concentration of the Chl-a measured by the spectrophotometer.

The influence of suspended solid particles to the Chl-a measurement was tested by adding different quantities of powder clay (by Vigorplant Italia) in 20 liters of MilliQ water containing a fixed concentration of Chl-a (312 µg/L). The fluorescence spectra of nine samples containing different quantities of clay (10 to 165 mg/L) were recorded in a rapid succession. The clay particles were kept suspended by mixing the whole sample with a PVC rod just before recording the measurement.

To assess the temperature effect on fluorescence signal, six freshwater samples (20 L each one) with different temperatures (from 4 °C to 32 °C), but with the same Chl-a concentration were prepared for the analysis. The temperature was measured by a total immersion Mercury-in-glass thermometer −10 °C to 110 °C × 1 °C, 250 mm length (by Brannan, Cumbria, UK) simultaneously with the fluorescence spectra recording.

In order to test the sensor capability on revealing substances different from Chl-a, a qualitative assessment was performed in laboratory for Cyanobacteria (*P. rubescens*). For this experiment, two samples of 10^5^ cells/mL and 10^6^ cells/mL were prepared using *P. rubescens* laboratory cultured. They were excited by blue light (450 nm LEDs), having a total power of 1 Watt.

Taking into account that the sensor poses a dual excitation wavelength, its capability on revealing diesel oil and protein-like material was assessed by employing UV light. The excitation sources were four LEDs emitting 280 nm and having a total power of 2.4 mW. One of the main fluorophores in protein-like material is tryptophan. For this, we used a tryptophan standard sample supplied by Sigma Aldrich. As oil and protein-like material may have similar fluorescence, three different samples were prepared for analysis and comparison: one sample of Milli-Q water containing tryptophan (a few mg/L), one containing diesel oil (1 drop/L) and one with both substances. We used as reference a sample of pure Milli-Q water.

### 3.2. Materials and Procedures for Field Tests

**Figure 10:**
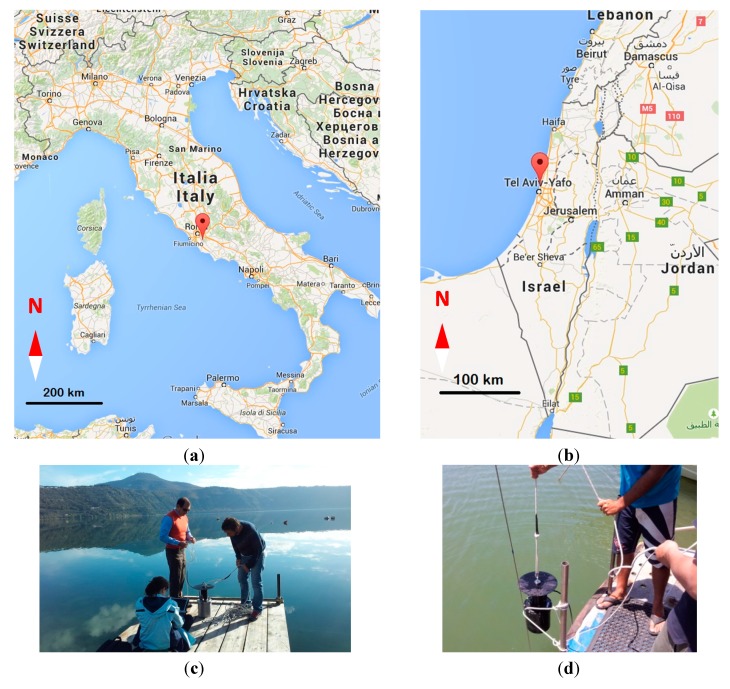
Location of the two sites where the sensor was tested and some pictures taken during the campaigns: (**a**,**c**) Albano Lake (Italy); (**b**,**d**) Herzliya Harbor (Israel).

Tracking and recording distribution or concentration changes of microscopic living plant matter (phytoplankton or algae) in marine habitat are of great interest to scientists, environmental researchers, ecologists, and natural and marine resource managers who have to implement conservation solutions and remedial actions. In this view, our team has conducted field campaigns at Albano Lake located in the Latium area, 20 kilometres in the southeast of Rome (Italy) and in Herzliya Harbor, a few kilometers North East of Tel Aviv (Israel) (Figure 10).

During the field campaign at Albano Lake, the sensor response was validated by comparing its result to the one given by a commercial spectrometer. For this purpose, samples of lake water were collected in bottles and brought immediately into the laboratory by keeping their temperature constant. The measurement of Chl-a concentration was achieved by the Perkin Elmer Lambda 25UV/VIS Spectrometer.

**Figure 11:**
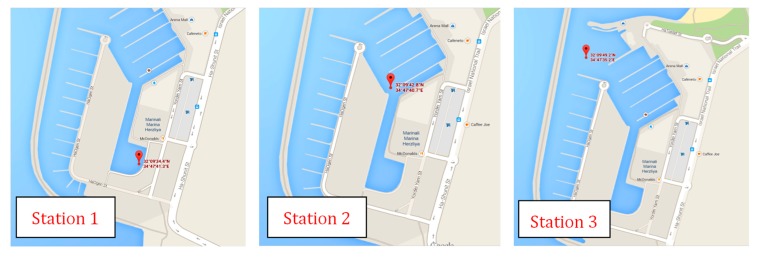
Geographical position of Stations 1, 2 and 3 in Herzliya Harbor.

In the Herzliya Harbor, three different points of the gulf were checked for the presence of Chl-a from a boat of the “Israel Oceanographic Research”. The stations where our sensor took measurements (about 20 spectra) are indicated in Figure 11.

## 4. Results and Discussion

### 4.1. Sensor Calibration with Chl-a

**Figure 12:**
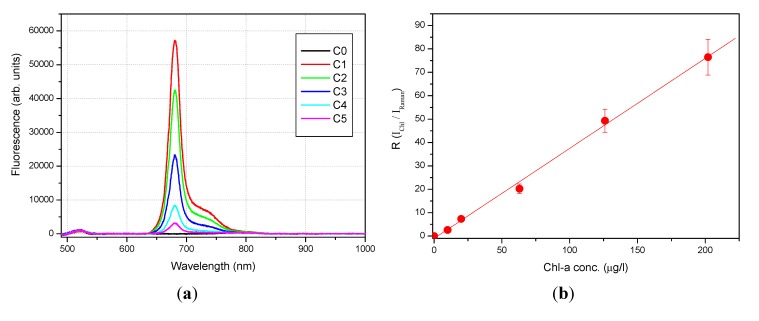
Laboratory calibration: (**a**) spectra of Milli-Q water (C0) and five different concentrations of Chl-a in Milli-Q water (C1—most concentrated to C5—less concentrated); (**b**) graphical representation of the calibration data.

The system has been tested and calibrated in laboratory in an arrangement simulating its deployment in water. Figure 12a shows the fluorescence spectra (background-subtracted) of Milli-Q water with five different concentrations of Chl-a ranging from 40 µg/L to 880 µg/L. The water Raman scattering is peaked at 535 nm as expected. The emission at 680 nm and 730 nm are coming from the Chl-a fluorescence. The pigment concentration is proportional to the ratio between the fluorescence emissions at 680 nm and 535 nm.

Figure 12b reports the graphical representation of the calibration data. The error bars are the standard deviation of the ratio R = I_chl_/I_Raman_. The ratio R represents the Chl-a concentration expressed in Raman units (RU), I_chl_ is the Chl-a fluorescence peak intensity, while I_Raman_ is the water Raman peak intensity. Linear regression of data indicated an intercept value of −1.004 ± 1.060, a slope value of 0.385 ± 0.011, a linear correlation coefficient r = 0.9985, and a coefficient of determination r^2^ = 0.9970. The calibration measurements were conducted at fix temperature (23 °C). Table 2 contains the results obtained for all six analyzed samples.

**Table 2:** Chl-a calibration results.

ample	I_chl_ (arb. units)	I_Raman_ (arb. units)	R = I_chl_/I_Raman_ (R.U.)	Chl-a Conc. (µg/L)
C0	0	1185 ± 103	0	0
C1	56749 ± 384	742 ± 95	76.49 ± 9.81	202
C2	42053 ± 323	853 ± 88	49.32 ± 5.09	126
C3	22895 ± 405	1131 ± 121	20.23 ± 2.19	63
C4	8250 ± 142	1122 ± 71	7.35 ± 0.48	20
C5	2938 ± 97	1126 ± 95	2.61 ± 0.23	10

Many scientific papers [12,13,14,15,16] have highlighted the importance of turbidity and temperature effects for *in situ* fluorescence measurements. For this reason, the influence of suspended clay particles and temperature quenching was explored in laboratory under controlled conditions.

**Figure 13:**
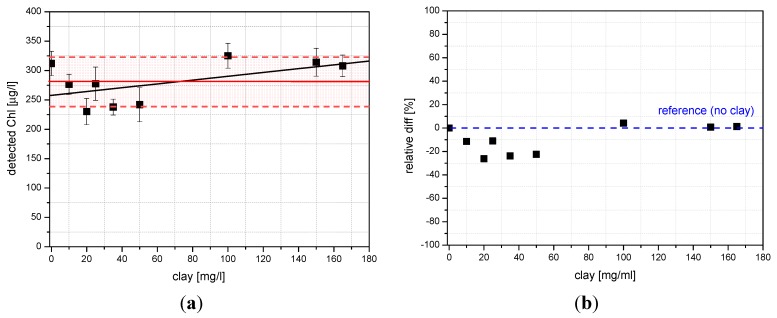
Clay interference on Chl-a detection (**a**); relative differences between the blank (sample without clay) and different turbid samples (**b**).

Figure 13a reports the results about clay interference on Chl-a measurements; the red continuous line represents the average value of Chl-a measured by the sensor after adding different quantities of clay, while Chl-a concentration is constant. As it can be noticed, the fluctuations of the Chl-a values are ranging within the interval defined by the calculated standard deviation (the red band) of nine different measurements. Also the linear fit of the measurements (the black line) falls within the red band. The linear regression of data indicated an intercept value of 257.71 ± 15.94, a slope value of 0.32 ± 0.19 and a linear correlation coefficient r = 0.5368. Thus, we find that there is no significant dependency of Chl-a value with respect to the quantity of the suspended particles, at least for the clay concentrations tested by us. This can be explained by the fact that our measurement is based on the ratio between the Chl-a fluorescence peak and the Raman water peak, which are probably influenced by the suspended particles in a similar way. Thus, their ratio is independent with respect to the presence of the suspended particles.

The relative differences between the reference (sample without clay) and other samples with added clay were plotted in the Figure 13b. The measurements fluctuate around the reference value within about 20%.

**Figure 14:**
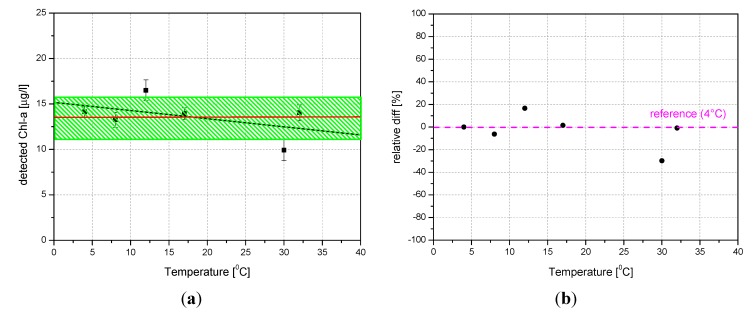
Effects of temperature on Chl-a detection (**a**); relative differences between the reference sample (4 °C) and samples of water containing a fixed amount of Chl-a at different temperatures (**b**).

Temperature effects on Chl-a fluorescence have been investigated in freshwater samples containing identical Chl-a concentrations. In Figure 14a, it can be seen that likewise in the case of clay interference, the fluctuations of the Chl-a values range within the interval defined by the calculated standard deviation (the green band) and the linear fit of the measurements (the black line) falls within the green band. This means that the Chl-a fluorescence and the water Raman peak are affected by the temperature in a similar way and their ratio can be considered temperature-independent. The linear regression of data produced an intercept value of 15.17 ± 1.62, a slope value of −0.09 ± 0.08 and linear correlation coefficient r = −0.4872.

Since the water density is at its maximum at 4 °C, we have considered it as our reference. In the Figure 14b we report the relative differences between the reference and the other five samples having temperatures ranging from 8 °C to 32 °C. The measurements fluctuate around the reference (4 °C) within about 20%.

The statistical error has been shown on the graphs (Figure 13a and Figure 14a) in order to show that the difference between each single measurement and the average value is not statistically significant. Error bars from Figure 13a and Figure 14a indicate the accuracy of the system in its present status: the relative error varies from 3% to 12%. Keeping in mind that the system was designed for a fast measurement of water quality to give an alert, this result turn to be satisfactory for that purpose. Taking into account that: (1) the fluctuations of the measurements are generally smaller than the standard deviation of the measurements themselves (see bands); (2) each measurement is statistically compatible within its error with the average of the measurements plus/minus the standard deviation (bands); (3) the linear fits of the measurements fall within the bands, we feel that any attempt to fit a non-linear curve to the data could be statistically meaningless.

**Figure 15:**
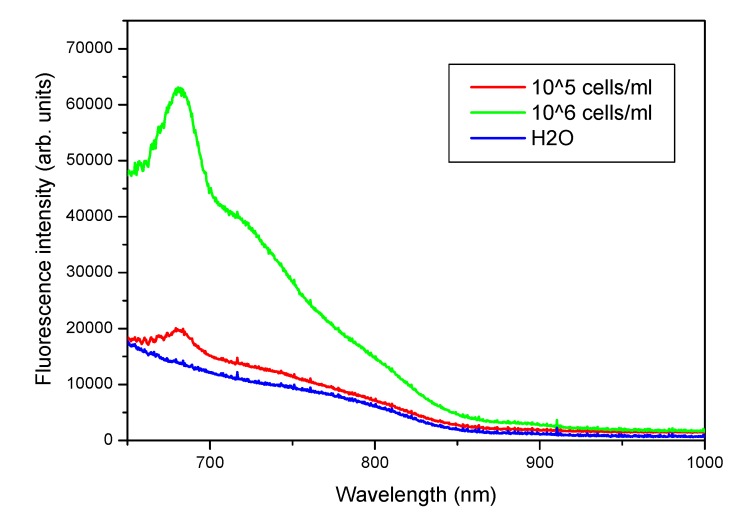
Cyanobacteria (*P. rubescens*) fluorescence spectra.

In order to test the sensor’s capabilities in terms of revealing substances different from Chl-a, a qualitative assessment was performed in laboratory for Cyanobacteria (*P. rubescens*). Figure 15 highlights the presence of the characteristic peak at 690 nm. We chose to test the sensor for cyanobacteria because they are microorganisms that can reproduce explosively under certain conditions, becoming harmful to other species due to the produced toxins (cyanotoxins). For this reason, it is important to continuously monitor their evolution by means of networks of sensors able to take real-time measurements at different depths.

Since the florescence yield can vary from one type of algae to another, for each type of algae to be monitored in the future the sensor needs to undergo a specific calibration procedure by simultaneously employing other measuring instruments.

### 4.2. Laboratory Tests on Diesel Oil and Tryptophan

Increasing anthropogenic dissolved organic matter (DOM) inputs from sewerage and farm wastes as well as oil pollution on the water surface demands a rapid pollution monitoring tool. Protein-like material and diesel oil may show similar fluorescence spectra, and this may pose some difficulties in discriminating between them. Thus, a qualitative assessment was performed in the laboratory for diesel oil and tryptophan fluorescence detection in water in order to put in evidence spectral differences that may lead to their identification, whether they are present simultaneously or individually in water.

**Figure 16:**
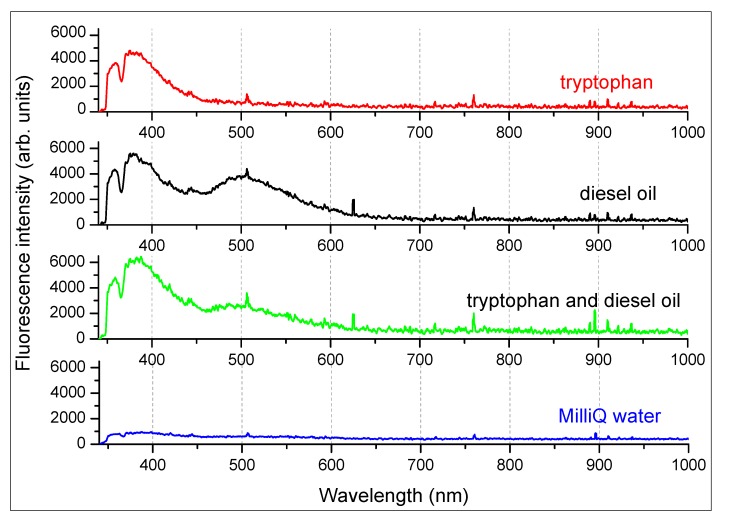
Diesel oil, tryptophan and Milli-Q water fluorescence spectra measured upon excitation at 280 nm.

Typical spectra of tryptophan and diesel oil in water are reported in Figure 16, which shows the spectral features characterizing the two substances in the 340–600 nm range. Milli-Q water was used as a reference and, as expected, does not possess any spectral feature in this region. From the comparison of the florescence spectra, it can be noticed that tryptophan and diesel oil share the emission peak at 380 nm, but diesel oil exhibit also a second peak at 500 nm. It is reasonable to assume that, in the case of diesel oil, the ratio between the peak centered at 380 nm and the one at 500 nm will not vary with the concentration. Thus, if tryptophan-like material and diesel oil are present in water simultaneously, the ratio between the two peaks will be higher and will increase proportionally with the tryptophan-like material concentration. This demonstrates that it is possible to detect their presence also in mixture. In more complex mixtures containing different compounds that exhibit similar fluorescence emissions, Parallel Factor Analysis (PARAFAC) could be applied in order to identify each single component [11].

### 4.3. Field Results

In Albano Lake (Italy) we measured the Chl-a fluorescence at the water surface and at 50 cm depth by submerging the sensor in water at a distance of about 10 m from the shore. The results of the experiment are reported in Table 3. Samples of water were also brought into the laboratory for Chl-a concentration measurement with the Perkin Elmer spectrometer apparatus, which provided a very good agreement with the values measured by the submersible sensor. In fact, the last column in the Table 3 indicates that the relative differences between the value measured in laboratory by the spectrometer and the estimated value obtained by the sensor are very small, confirming that the sensor is working well. Out of caution, we shall consider the accuracy of the sensor the value indicated by the relative statistical error E_R_.

**Table 3:** Results of Albano Lake measurements achieved by the submersible spectrofluorometer.

Parameter	Chl-a (RU)	E (RU)	E_R_ (%)	Chl-a (µg/L)	Relative Diff. (%)
Lake surface	0.0536	0.0010	1.9	0.181	+0.03%
50-cm depth	0.0737	0.0021	2.8	0.249	−0.02%

The measurements indicated that a Chl-a concentration of about 0.181 µg/L of was present at the lake surface, while at 50 cm depth the concentration was 0.249 µg/L. From these results we can conclude that if the sensor was able to detect approximately 0.2 µg/L with a relative error of about 2%–3%, as a consequence, it could be able to detect lower quantities, in the order of 0.01 µg/L.

During the campaign conducted in the Herzliya Harbor, we measured Chl-a in three different stations. In Station 1 measurements were carried out both in the morning and in the afternoon. At this site we recorded a higher Chl-a concentration in the morning (AM) than in the afternoon (PM). Regardless, water in the harbor was highly eutrophic all day long and Chl-a concentrations were extremely high. Their values are summarized in Table 4. As expected, the Chl-a concentration is lower at the exit of the harbor, where its waters mix with those of the open sea.

**Table 4:** Chl-a concentrations measured in the Herzliya Harbor.

Station	GPS Position	Depth	Chl-a (µg/L)
1 (AM)	N32°09′ 0.5728, E034º47′ 0.6879	1 m	736
1 (PM)	N32°09′ 0.5728, E034º47′ 0.6879	surface	309
2	N32°09′ 0.7129, E034º47′ 0.6784	surface	687
3	N32°09′ 0.8204, E034º47′ 0.5874	surface	43

## 5. Conclusions and Future Developments

Monitoring the phytoplankton population and its distribution is essential for assessing water composition, health and ecological status. Therefore, we addressed the need for fast and real-time tracking of some important indicators of the ecological status of natural waters by developing an innovative submersible spectrofluorometer suitable for monitoring Chl-a, oil and protein-like material at different depths. The optomechanical part of the apparatus followed a conceptual design that avoided filters and pumps while maintaining a high signal-to-noise ratio. By using LEDs as an excitation source, it was possible to develop a compact system with low power consumption and low costs. Being fully automated, one measurement takes less than one minute. The main advantage of the sensor over the existing state of the art is its unrivalled flexibility in different application scenarios and accuracy for many water components, due to the availability of the full spectrum, which allows for detection of many other pollutants and pigments. Thus, it can be deployed in various water bodies with different ecological statuses.

The sensor was calibrated and tested during laboratory experiments as well as in field campaigns conducted in Albano Lake, Italy and Herzliya Harbour, Israel. The achieved sensitivity for Chl-a detection was found to be at least 0.2 µg/L. The influence of temperature and suspended solid particles was assessed upon Chl-a laboratory measurements. The use of water Raman peak as a reference turned out to be very useful to override the fluorescence quenching and matrix effects. During laboratory tests, we also applied the sensor for detection of microorganisms such as cyanobacteria.

In spite of the low excitation power of the adopted UV LEDs, the sensor was confirmed to be appropriate for detecting diesel oil and tryptophan-like material as well.

In conclusion, the sensor proved to be suitable to detect Chl-a, Cyanobacteria, diesel oil and protein-like material and could therefore be employed for monitoring oil pollution, harmful algal bloom, water drinking quality and marine environment.

In the next oceanographic campaign, the use of a CTD sonde and a lidar fluorosensor together with the developed sensor is foreseen in order to assess temperature, conductivity, depth, and extinction coefficient. We plan to measure diesel oil, different algal strains and organic material by simultaneously employing other measuring instruments useful for calibration and cross correlation.

In the future, the sensor can be further improved by developing a chamber able to resist deeper depths as well as by using LEDs with different emission wavelengths. Moreover, a network of submersible spectrometers which communicate and transfer measurements by optical wireless links to the sea surface for teleprocessing by an analyzing center can be developed. The analyzing center may also receive information from the satellite and fuse it with the spectrometer network information in order to augment and expand the data and get hyper-precise results.
